# Ascending with ultrasound: telementored eFAST in flight—a feasibility study

**DOI:** 10.1007/s10140-023-02186-x

**Published:** 2023-12-09

**Authors:** Peder Christian Engelsen, Fridtjof Heyerdahl, Dharani Dhar Maddali, Mehdi Sadat Akhavi, Ragnhild Marie Undseth, Ole Jakob Elle, Henrik Brun

**Affiliations:** 1 The Intervention Centre, Oslo University Hospital, Oslo, Norway; 2 The Air Ambulance Department, Oslo University Hospital, Oslo, Norway; 3 Institute of Clinical Medicine, University of Oslo, Oslo, Norway; 4 Department of Informatics, University of Oslo, Oslo, Norway

**Keywords:** Ultrasound, Telementoring, eFAST, Helicopter, Prehospital medicine, Feasibility

## Abstract

**Purpose:**

Teleultrasound uses telecommunication technologies to transmit ultrasound images from a remote location to an expert who guides the acquisition of images and interprets them in real time. Multiple studies have demonstrated the feasibility of teleultrasound. However, its application during helicopter flight using long-term evolution (LTE) for streaming has not been studied. Therefore, we conducted a study to examine the feasibility of teleultrasound in an Airbus H145 helicopter.

**Methods:**

Four anesthesiologists and one military physician were recruited to perform telementored extended Focused Assessment with Sonography in Trauma (eFAST) during nine helicopter flights, each with a unique healthy volunteer. A radiologist was recruited as a remote expert, guiding the physicians in their examinations. The examining physicians reported the user experience of telementored eFAST on a questionnaire, while the remote expert rated the diagnostic quality of the images on a 1–5 Likert scale. In addition, we measured the duration of the examinations and key LTE network parameters including signal strength, quality, and continuity.

**Results:**

The images were rated to an average of 4.9 by the remote expert, corresponding to good diagnostic quality. The average duration of telementored eFAST was 05:54 min. LTE coverage was negatively affected by proximity to urban areas and ceased above 2000 ft altitude. Occasional audio problems were addressed by using the Voice over LTE network for communication. The examining physicians unanimously reported on the questionnaire that they would use telementored eFAST on patients.

**Conclusion:**

Telementored eFAST is feasible in ambulance helicopters and can produce images of good diagnostic quality. However, it relies on stable LTE coverage, which is influenced by many factors, including the helicopter’s altitude and flight path. Furthermore, its benefit on patient outcomes remains to be proven.

**Graphical abstract:**

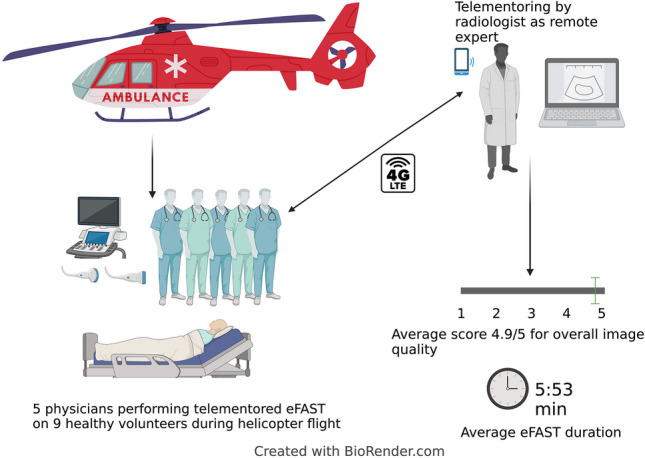

**Supplementary Information:**

The online version contains supplementary material available at 10.1007/s10140-023-02186-x.

## Introduction

Focused Assessment with Sonography in Trauma (FAST) has improved the assessment and management of trauma patients [1]. In prehospital settings, FAST has significantly reduced the time for patients to receive critical care [2]. Portable devices have expanded the reach of emergency ultrasound to extreme prehospital environments such as combat zones and spaceflight [3–5]. However, correct interpretation of ultrasound images is operator-dependent, and training in emergency ultrasound can be challenging due to the sporadic exposure to acute conditions such as hemoperitoneum and pneumothorax [6, 7].

Consequently, there is great interest in teleultrasound where remote experts provide real-time support to novice operators in image acquisition and analysis. In this study, “telementoring” refers to the process where a remote expert guides the on-site operator in obtaining clinically meaningful images.

Teleultrasound can assist prehospital care providers in diagnosing life-threatening conditions, such as intraperitoneal hemorrhage and pneumothorax. Early diagnosis of these conditions expedites life-saving interventions and ensures that patients are directed to the appropriate level of care. Although numerous studies have demonstrated the feasibility of teleultrasound, further research is needed to optimize transmission systems, particularly for examinations on moving platforms [8].

Reliable networks are essential for prehospital teleultrasound. In our study, we used long-term evolution (LTE) for image transmission. The power and quality of the LTE signal can be gauged by Reference Signal Received Power (RSRP) and Reference Signal Received Quality (RSRQ), respectively. Signal-to-Interference-plus-Noise Ratio (SINR) further quantifies signal quality, with higher values indicating better reliability. Connection stability can also be monitored by tracking changes in cellular ID, an identifier unique to each station in an LTE network [9].

While the diagnostic accuracy of extended FAST (eFAST) in-flight has been studied [10], its in-flight application supported by telementoring remains unexplored. In this study, we sought to bridge this knowledge gap and explore key aspects of telementored eFAST performed during flights at altitudes of 500 and 1000 ft.

## Methods

### Participants and preparations

Nine medical students were recruited after providing informed consent. Our exclusion criteria were BMI > 30 and aerophobia. Five physicians were recruited to perform telementored eFAST. This group included four anesthesiologists from the Air Ambulance Department at Oslo University Hospital (OUH) and one military physician with experience in emergency medicine. A radiologist with 12 years of experience with abdominal and emergency ultrasound was recruited as the remote expert.

The anesthesiologists were given a 15-min presentation on the fundamental principles of the eFAST procedure before the flight. To ensure unambiguous communication during telementoring, the remote expert and examining physicians were provided with an illustration of anatomical planes and standardized probe movements such as tilting, sliding, and rotation. On the flight day, the latter group performed eFAST once on a healthy volunteer to get accustomed to the ultrasound device.

### Setup

A five-blade rotor Airbus H145 helicopter dedicated to research purposes was used for the study [11]. The helicopter was manned by a pilot, paramedic, physician, data collector, and one healthy volunteer. To reduce network disturbances, we followed a pre-planned route near Oslo, Norway, covering both sea and inland regions. During the examinations, the helicopter maintained a stable altitude and avoided urban areas.

**Fig. 1:**
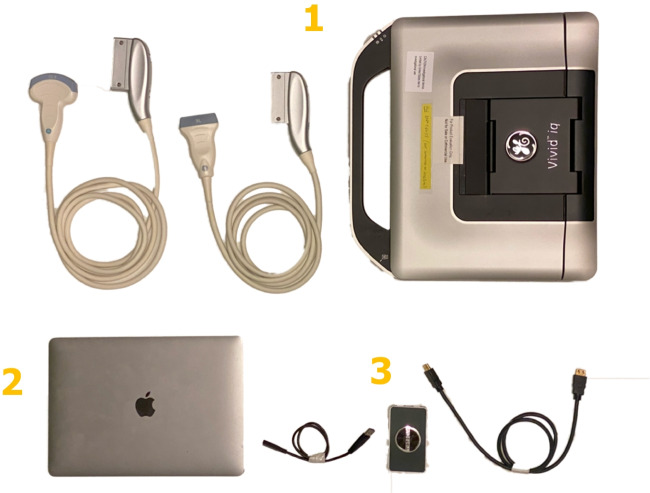
Onsite teleultrasound setup, detailed information provided in Table 1

Onboard thehelicopter, we connected a laptop computer (MacBook Air M1 2020) to a local Wi-Fi, which in turn linked to the LTE network (Table 1) (Fig. 1). This setup allowed for both the sharing of ultrasound images through video conferencing software (Whereby) and two-way audio communication. The physicians communicated with the remote expert using a Bluetooth headset (Bose A20) (Table 2).

During flight, the healthy volunteers were examined with eFAST in a supine position on a stretcher. A commercial ultrasound device (GE Vivid IQ) was used for the examination and connected to the laptop computer through a USB video grabber (Magewell Capture Card).

**Table 1:** Onsite teleultrasound setup

	Function	Name	Producer
1	Ultrasound system	C1-5-RS curved array probe 1.5–5 MHz	GE Healthcare, Chicago, Illinois, USA
		12L-RS linear probe 6–13 MHz	
		Vivid IQ	
2	Onsite laptop (image streaming)	MacBook Air, Apple M1, 16 GB RAM, macOS Ventura (version 13.0.1)	Apple, California, USA
3	USB video grabber	USB Capture HDMI 4K Plus	Magewell, Nanjing, Jiangsu, China

### Data collection

Anthropometric data (height, body weight, and age) were collected for the healthy volunteers. We gauged the ultrasound experience of the anesthesiologists and the military physician with a POCUS course survey from the University of Utah (Appendix 1).

We used a modified version of a questionnaire (Appendix 2) from the F.A.S.T.E.R. trial by Mazur et al. to gather feedback [12]. The remote expert rated the ultrasound images on a Likert scale from 1 to 5, where 1 corresponded to no diagnostic utility and 5 corresponded to good diagnostic quality. The examining physicians provided feedback on their user experience during the procedure.

The duration of the examination was defined by the time spent from the moment the activated probe was placed on the skin until the physician and remote expert agreed that the examination was complete. We measured the duration of eFAST performed by a second radiologist on healthy volunteers in a controlled hospital setting and compared this to the in-flight telementored eFAST duration. To validate our method for assessing image quality, this radiologist also reviewed screen-recorded ultrasound images from the flights using the same questionnaire as the remote expert.

A combined satellite and cellular system (Flightcell DZMx) tracked key network parameters such as RSRP, RSRQ, and SINR, as well as the cellular ID of the closest base station, altitude, and GPS coordinates.

The primary outcome of this study was the diagnostic quality of the eFAST images as rated by the remote expert, comparing the images from examinations at 500 and 1000 ft. This pertains to the ability of the images to provide clinically relevant information rather than their pure resolution or transmission quality.

**Table 2:** Remote setup and in-flight equipment

Function	Name	Producer
Video conferencing software	Whereby	Video Communication Services AS, Måløy, Vestland, Norway
Remote laptop (image streaming)	MacBook pro 2,4 Ghz Intel Quad Core, 8 GB RAM, Ventura (version 13.0.1)	Apple, California, USA
Headsets	A20 Aviation Headset with Bluetooth	Bose Corporation, Massachusetts, USA
	H10-13 H Aviation Headset	David Clark Company, Worcester, Massachusetts, USA
Satellite and cellular system	Flightcell DZMx	Flightcell International Limited, Nelson, New Zealand
Helicopter	H145	Airbus Helicopters SAS, Marignane, France

The secondary outcomes were the user feedback on the questionnaires, duration of the examinations, and connectivity of the LTE network measured with RSRP, RSRQ, and SINR and the handover rate. The latter represents the frequency of changes in the cellular ID over a 5-min interval.

### Statistical analysis

Statistical computations were executed in Python and Microsoft Excel. Correlation analysis, specifically the Pearson correlation coefficient, was used to investigate the relationship between altitude and RSRQ values. Differences in image ratings were assessed with paired and independent sample *T*-tests, with the significance level set at 0.05 (Table 3).

**Table 3:** Test subject demographic and self-reported POCUS experience

Age (mean, SD)	26 (±2,9)
BMI (mean, SD)	24,1 (±2,8)
Male (number, %)	3 (33,3 %)
Female (number, %)	6 (66,7 %)
Physicians, years in practice	15.4 (±5.5)
Years of POCUS experience (median, IQR)	11 (6)
Able to acquire and interpret images with POCUS	
Neutral	1
Agree	3
Strongly agree	1
Confident in performing eFAST	
Disagree	1
Neutral	3
Agree	1

## Results

From November 16th to November 17th, 2022, nine healthy volunteers (six women and three men) between the ages of 22 and 32 were examined with telementored eFAST during ﻿flight (Table 3). The weather was cloudy with an average temperature of 2.2 °C, and the strongest winds recorded were 9.4 m/s.

Ultrasound images were successfully obtained and analyzed in all projections from the nine volunteers. One examination was interrupted at 500 ft due to loss of connection with the remote expert. We made an ascent to 1000 ft, where the connection was reestablished, and continued the examination.

The remote expert rated the diagnostic quality of the images on average at 4.9 (Table 4), with no significant difference in quality between the altitudes of 500 and 1000 ft (*P* > 0.05). The image quality in the right upper quadrant (RUQ) was significantly higher compared to the left upper quadrant (LUQ) (*P* < 0.05).

**Table 4:** Remote expert assessment of eFAST image quality

	500 ft	1000 ft	Pooled
RUQ image quality	4.6 (± 0.5)	4.6 (±0.1)	4.6 (± 0.5)
LUQ image quality	4.1 (± 0.8)	4.1 (± 0.8)	4.1 (± 0.1)
Suprapubic image quality	4.9 (± 0.4)	4.9 (± 0.3)	4.9 (± 0.3)
Pericardium image quality	4.4 (± 0.5)	4.4 (± 0.7)	4.4 (± 0.6)
Lung ultrasound image quality	5.0 (± 0.0)	5.0 (± 0.0)	5.0 (± 0.0)
Overall image quality	4.9 (± 0.1)	5.0 (± 0.0)	4.9 (± 0.3)

According to the questionnaire responses, all five examining physicians found the remote expert’s instructions clear and were confident in performing telementored eFAST on patients during flight.

**Fig. 2:**
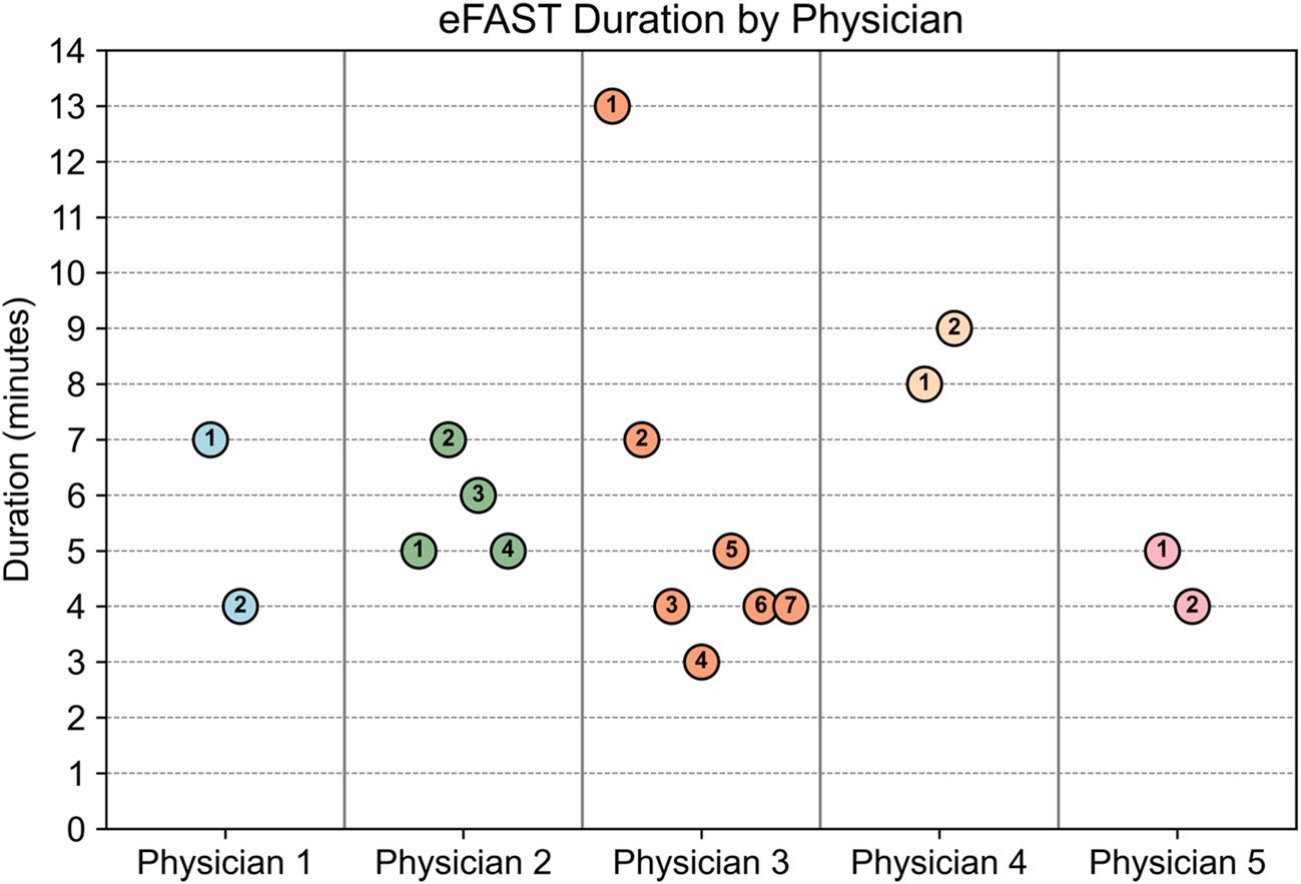
Dot chart displaying the duration of telementored eFAST conducted by the five physicians. Each dot represents an examination, its *y*-position indicates duration, its color denotes the physician, and the number within represents the order in which the examinations were conducted

The average duration of the examination was 5:54 min (SD 2:27 min) for the physicians and 2:53 min (SD 1:08 min) for the radiologist performing eFAST on the same healthy volunteers in a hospital setting (Fig. 2).

Table 5 compares image quality ratings from the remote expert and the second in-hospital radiologist. Complete screen recordings were only available for three of the eFAST examinations. These recordings served as the basis for our comparative analysis. The agreement on the binary outcomes was 100%, but the radiologist’s overall rating was lower than the remote expert’s (*P* < 0.001).

**Table 5:** Comparison of image quality ratings

Radiologist 1, remote expert		Radiologist 2, hospital setting	
Mean	Std Dev	Mean	Std Dev
4.65	0.58	3.81	0.8

**Fig. 3:**
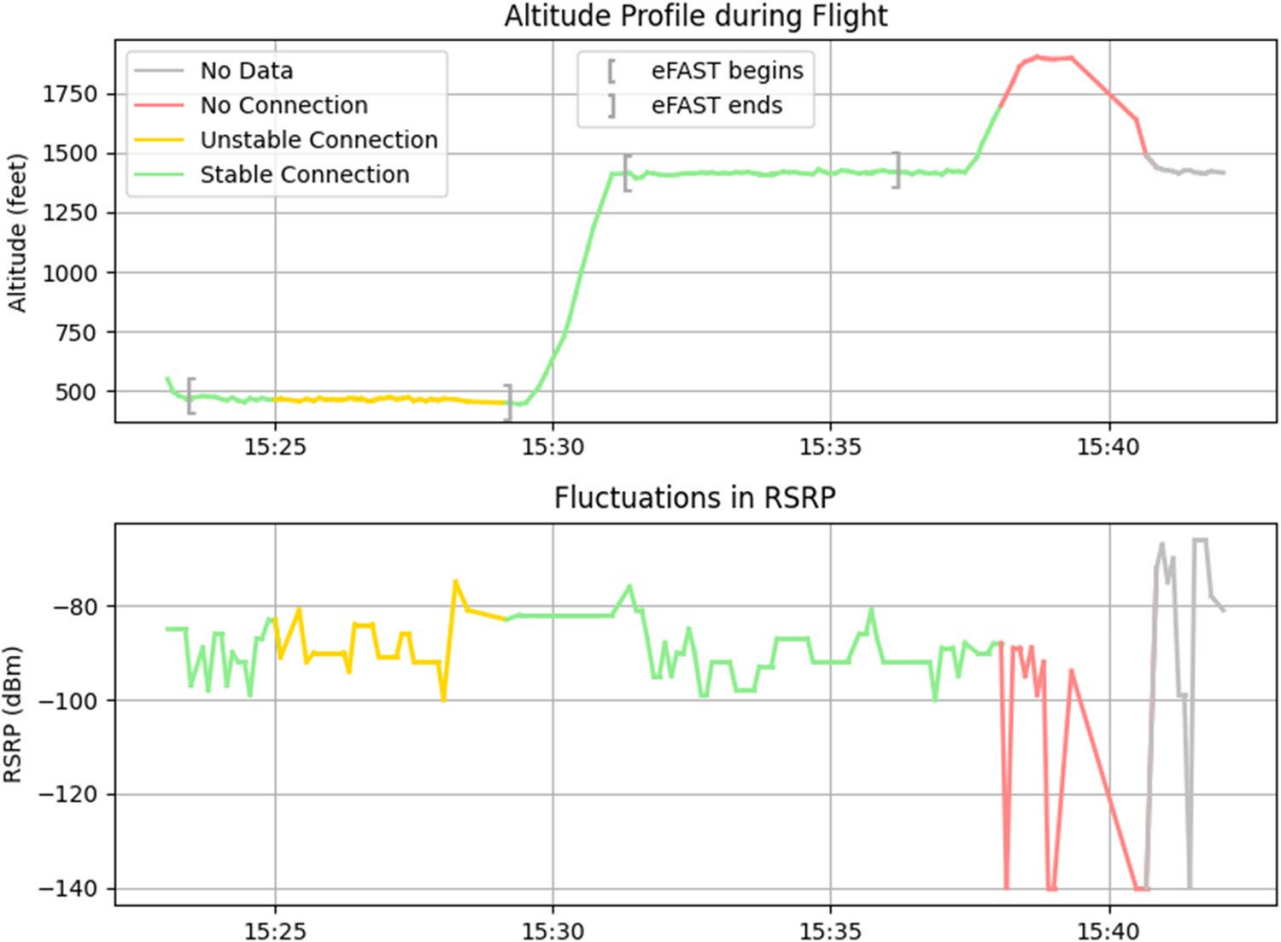
Altitude and RSRP Signal Strength Plots. The top plot illustrates the altitude in feet over time, while the bottom plot represents the RSRP signal strength in dBm over the same time period. The colors indicate the connection status as experienced by the flight crew

We experienced difficulties with video conferencing in urban areas, where the mean handover rate was nearly double (16.2 ± 4.5) that of rural areas (8.6 ± 4.0). Moreover, the median SINR was significantly (*P* < 0.001) lower in urban areas (−5.8, IQR: 6.0) compared to rural areas (−1.4, IQR: 7.6). Network stability was also influenced by altitude extremes, with increased LTE coverage at ground level and loss of connection at 2000 ft (Fig. 3). An inverse correlation (−0.33 ± 0.02) between altitude and RSRQ further substantiates these findings.

**Table 6:** Reported technical problems

		Comments
No connection	1 out of 9 flights	Interruption at 500 ft altitude, connection reestablished at 1000 ft
Unstable connection	3 out of 9 flights	Frozen images, lag, brief losses of connection
No sound	3 out of 9 flights	Unreliable audio connection through laptop and video conferencing, telephone communication through Voice over LTE network found to be more reliable

In three flights, we resorted to an alternative method of communication due to audio problems (Table 6). The remote expert provided instructions via the chat function on the video conferencing software, which were read to the physician on the helicopter’s intercom. For the subsequent flights, we used the Voice over LTE network to contact the remote expert on his mobile phone. The physicians communicated with him through a headset (David Clarke H10-13), which provided a reliable audio connection.

## Discussion

This study adds to the existing body of evidence on the feasibility of teleultrasound by demonstrating its successful implementation in the challenging environment of helicopter flight. The physicians produced images of good diagnostic quality in all nine flights. The disparity in image quality between the RUQ and LUQ, with the former exhibiting superior quality, is consistent with findings reported in previous research [13]. This could be due to the spleen’s smaller size and the complexity of LUQ imaging compared to the more straightforward liver-centered RUQ imaging [14].

Image ratings were generally lower from the second in-hospital radiologist compared to the remote expert. Absence of live interaction might have contributed to lower ratings from the former, while novelty bias could have led to inflated ratings from the remote expert. Nevertheless, the agreement on binary outcomes, such as clear imaging of the pleura, suggests all the images met basic requirements.

Our study, unlike conventional teleultrasound studies, involved practitioners with prior experience in emergency ultrasound, including eFAST. The questionnaire data indicates that they still would incorporate telementored eFAST into actual patient care. This suggests the method’s applicability beyond the scope of novice practitioners.

The combination of standardized terms, a slideshow presentation, and the physicians’ existing familiarity with eFAST appears to have prevented misunderstandings of ultrasound terminology. The absence of reported difficulties in understanding the remote expert supports the emphasis placed on the use of shared ultrasound terminology in previous studies [15] .

The duration of eFAST was significantly longer in the helicopter compared to the hospital setting. In their regular practice, the anesthesiologists mainly performed the cardiac, lung, and procedural ultrasound for vascular access, unlike eFAST, which is a more extensive evaluation of several regions. Their varying and limited experience with eFAST, along with the challenging environment in-flight, likely extended the duration of the procedure and contributed to the large standard deviation of 2:27 min.

The impact of geographical location on connection stability emerged as an important finding in our study, but the reasons for this phenomenon are not fully understood. In urban areas, there are numerous pedestrian-focused base stations. A fast-moving helicopter in this environment is susceptible to interference and will trigger frequent handovers between base stations. As per the user feedback, SINR and handover rates, the proximity to urban areas seemed to have a more detrimental effect on connection stability than the variations in altitudes of 500 and 1000 ft. This highlights the importance of considering geographical factors in the planning and execution of teleultrasound procedures during flight.

However, our data indicate that altitude levels still play a role in determining LTE coverage and connection stability. Higher altitudes were inversely correlated with signal quality, and an ascent to 2000 ft resulted in immediate loss of connection. Thus, altitude should also be considered for ensuring optimal connectivity for in-flight teleultrasound.

In The Norwegian Air Ambulance Service, helicopters generally operate within an altitude range of 0–10,000 ft above mean sea level, usually staying below 5000 ft above ground level. The selection of flight routes and altitudes is a complex decision-making process influenced by a myriad of factors including weather, topography, tactical, and medical considerations. Pilots have the discretion to alter pre-planned altitudes and routes, given that such changes are permissible by weather conditions, the landscape, and air traffic control. However, any modifications aimed at enhancing teleultrasound communication must be weighed against potential delays in patient care and loss of flight time.

Communication between the physician and the remote expert on the Voice over LTE network outperformed communication on the video conferencing software with LTE connection. The latter included multiple wireless links which could compromise the reliability of the communication. In Voice over LTE, data packets containing audio transmission are prioritized to ensure a stable audio link. Users reported greater satisfaction with dependable audio communication, emphasizing its indispensability in telementored ultrasound.

An unforeseen yet enlightening event took place during our study. On the same day as the telementored examinations, an anesthesiologist from the study performed eFAST on a severely injured patient during helicopter flight. He reported that the prior telementoring effectively prepared him for this real-life application. This incident corroborates studies that demonstrate the value of teleultrasound as an educational tool [16].

Although teleultrasound shows promise, it is crucial to note that the technology does not negate the need for skilled health care personnel. In fact, it introduces an additional layer of complexity by demanding support from a remote expert. While the feasibility of teleultrasound has been demonstrated in emergency medicine, its clinical benefit for patients has not been established [8].

## Limitations

This study has several limitations. The sample size was small and consisted of young medical students, limiting the generalizability of our findings. The helicopter’s preplanned routes at 500 and 1000 ft, known to have stable LTE network coverage, may not reflect real-world conditions where signal stability can fluctuate. Additionally, audio problems forced alternative communication methods in three of the flights, introducing an element of inconsistency in our data.

## Conclusion

Telementored eFAST is feasible in ambulance helicopters, generating images of high diagnostic quality within an acceptable timeframe. Proper preparation and usage of shared terminology appeared to streamline the communication between the physicians and the remote expert. Factors such as altitude and flight location impacted connection stability. The benefit of teleultrasound for patients has yet to be proven.

### Supplementary information

ESM 1 (PDF 254 kb)

ESM 2 (PDF 252 kb)
