# Correction to: Ascending with ultrasound: telementored eFAST in flight—a feasibility study

**DOI:** 10.1007/s10140-024-02265-7

**Published:** 2024-07-20

**Authors:** Peder Christian Engelsen, Fridtjof Heyerdahl, Dharani Dhar Maddali, Mehdi Sadat Akhavi, Ragnhild Marie Undseth, Ole Jakob Elle, Henrik Brun

**Affiliations:** 1 The Intervention Centre, Oslo University Hospital, Oslo, Norway; 2 The Air Ambulance Department, Oslo University Hospital, Oslo, Norway; 3 Institute of Clinical Medicine, University of Oslo, Oslo, Norway; 4 Department of Informatics, University of Oslo, Oslo, Norway; 5 Department of Pediatric Cardiology, Oslo University Hospital, Oslo, Norway

**Correction to: Emergency Radiology (2024) 31:25–31**

10.1007/s10140-023-02186-x

The original article contains an error. The author Peder Christian Engelsen should also include affiliation 3 “Institute of Clinical Medicine, University of Oslo, Oslo, Norway”. The last author Henrik Brun added the address “Department of Pediatric Cardiology, Oslo University Hospital, Oslo, Norway”

The original article has been corrected.

