# Supplementary material for: Ascending with ultrasound: telementored eFAST in flight—a feasibility study
Source: Emerg Radiol. 2023 Dec 9;31(1):25–31. doi: 10.1007/s10140-023-02186-x (PMC10830595; doi:10.1007/s10140-023-02186-x)
Supplement: Supplementary file 1 — (PDF 254 kb) [file 10140_2023_2186_MOESM1_ESM.pdf]

Supplementary Material 1. 2018 POCUS course survey

POINT-OF-CARE-ULTRASOUND (POCUS) PRE-COURSE QUESTIONNAIRE

This questionnaire will be used for quality improvement and/or research purposes to improve point-of-care-ultrasound education. The questionnaire will be administered today, with an electronic follow-up questionnaire at 3 months, 6 months, and 12 months. It will take approximately 3-5 minutes to complete this questionnaire and approximately 2-4 minutes to complete each follow-up questionnaire (total time commitment 10-15 minutes). All responses will be kept confidential. Any quality improvement or research that is presented or published will be de-identified and anonymous. Thank you for your participation.

1. Specialty or location of work?
    a. PICU
    b. NICU
    c. PEM
    d. Other (specify): _____________

2. Role:
    a. MD, DO
    b. RN
    c. PA
    d. NP
    e. Other (specify): ______________

3. Years in practice: _______________

4. Where do you currently practice: ________________________

5. On average, how many patients do you care for in a week?
    a. <10
    b. 11-20
    c. 21-30
    d. ≥ 31
    e. 0ther:

6. What prior training do you have with Point of care ultrasound or POCUS? Please list everything and details
    a. Medical school
    b. Residency training
    c. Fellowship training
    d. Conferences:
    e. Simulation labs
    f. Online courses:
    g. Websites:

   h. Self-taught
   i. Other: _________________

7. How many years of experience do you have with POCUS? __________

8. Do you have an ultrasound machine in your department that you can use for POCUS?
   a. Yes
   b. No
   c. Not sure

9. Do you have an ultrasound medical director in your department that can provide leadership and education to help you acquire and or maintain POCUS skills?
   a. Yes
   b. No
   c. Not sure

10. Do you have a POCUS curriculum within your department that: (select all that apply)?
    a. Can educate and train residents/fellows to use POCUS
    b. Can educate and train attending physicians to use POCUS
    c. Can educate and train advanced practice providers to use POCUS
    d. Can educate and train nurses to use POCUS
    e. Provides a credentialing pathway for untrained POCUS users
    f. Provides a credentialing pathway for experienced POCUS users
    g. Has a CME pathway to maintain credentialing?
    h. Has a quality assurance program to review scans, problem solve and provide ongoing training

11. Approximately how many patients have you scanned in the last month:
    a. 0
    b. 1-2
    c. 3-4
    d. >4

12. Approximately how many patients have you scanned in the last 3 months:
    a. 0
    b. 1-3
    c. 4-7
    d. >7

13. Approximately how many patients have you scanned in the last 6 months:
    a. 0
    b. 1-4
    c. 5-8
    d. 9-12

e. >12

14. Approximately how many patients have you scanned in the last 12 months:
    a. 0
    b. 1-5
    c. 6-10
    d. 11-15
    e. >15

15. What scans do you typically perform?
    a. E-FAST
    b. Focused cardiac
    c. IVC volume assessment
    d. Lung
    e. Soft Tissue
    f. Procedural
    g. Other (specify): _______________________

16. What are the barriers that keep you from performing POCUS? (select all that apply)
    a. Lack of a machine
    b. Lack of an ultrasound medical director
    c. Lack of an ultrasound pathway/curriculum to obtain and maintain credentialing for fellows and attending physicians
    d. Lack of department or administrative/institutional support
    e. Lack of time
    f. Lack of confidence in your ability to obtain images
    g. Lack of confidence in your ability to interpret images
    h. Lack of a quality assurance program to verify image acquisition and interpretation
    i. Lack of national norms/guidelines to establish diagnostic ultrasound credentialing thresholds
    j. Lack of experienced faculty (internal or external) for hands-on training and maintenance education
    k. Concern for inter-discipline conflict (cardiology or radiology) regarding clinical use of diagnostic ultrasound in practice
    l. Lack of a credentialing process
    m. Other: ____________________________________

17. What scans would you like to learn and perform? _____________________________

18. What could be done to help you personally use POCUS? ________________________

19. What suggestions or feedback do you have to help you or your colleagues use POCUS?
____
____
____

For each of the following statements, please circle the number that best corresponds to your response:

	Strongly Disagree	Disagree	Neutral	Agree	Strongly Agree
20. I am confident adjusting the basic knobs such as gain and depth	1	2	3	4	5
21. I am confident at choosing the correct probe for a given patient and purpose	1	2	3	4	5
22. I am confident finding and obtaining vascular access with POCUS	1	2	3	4	5
23. I am confident at performing either a FAST exam and/or an EFAST exam	1	2	3	4	5
24. I am confident that I can diagnose pericardial effusion with POCUS	1	2	3	4	5
25. I am confident that I can diagnose tamponade with POCUS	1	2	3	4	5

26. I am confident that I can diagnose a pneumothorax with POCUS	1	2	3	4	5
27. I am confident that I can diagnose fluid in the peritoneum such as hemoperitoneum or ascites with POCUS	1	2	3	4	5
28. I am confident in my ability to obtain basic cardiac views with POCUS	1	2	3	4	5

	Strongly Disagree	Disagree	Neutral	Agree	Strongly Agree
29. I am confident in my ability to assess left ventricular function with POCUS	1	2	3	4	5
30. I am confident in my ability to perform a thoracic examination with POCUS	1	2	3	4	5
31. I am confident in my ability to use POCUS for diagnostic neonatal applications	1	2	3	4	5
32. I am confident in my ability to evaluate right ventricle function and volume overload with POCUS	1	2	3	4	5
33. I am confident in my ability to evaluate volume responsiveness with POCUS	1	2	3	4	5

34. I am confident in my ability to diagnose shock with POCUS	1	2	3	4	5
35. I am confident in my ability to perform neonatal procedures with POCUS	1	2	3	4	5
36. I am confident in my ability to acquire images and interpret them with POCUS "putting it all together"	1	2	3	4	5

POINT-OF-CARE-ULTRASOUND (POCUS) POST-COURSE EVALUATION

For each of the statements below, please circle the number that best characterizes your response:

	Strongly Disagree	Disagree	Neutral	Agree	Strongly Agree
1. I was satisfied with the course didactics	1	2	3	4	5
2. I was satisfied with the hands-on learning modules	1	2	3	4	5
3. In general, I was satisfied with the course instructors	1	2	3	4	5

	Physician no.	1	2	3	4	5
1	Years in practice	21	17	13	20	6
2	On average, how many patients do you care for in a week?	<10	11-20	21-30	11-20	<10
3	What prior training do you have with Point of care ultrasound or POCUS? Please list everything and details					
a.	Medical school					
b.	Residency/fellowship training			✓	✓	
c.	Conferences		✓	✓		✓
d.	Simulation labs		✓	✓	✓	✓
e.	Online courses		✓	✓		✓
f.	Websites	✓	✓	✓	✓	✓
g.	Self-taught	✓	✓	✓	✓	✓
i.	Other	Colleagues	✓			
4	How many years of experience do you have with POCUS?	10	11	13	15	6
5	Do you have an ultrasound machine in your department that you can use for POCUS?	Yes	Yes	Yes	Yes	Yes
6	Approximately how many patients have you scanned in the last month:	>4	>4	>4	>4	3-4
7	Approximately how many patients have you scanned in the last 3 months:	>7	>7	>7	>7	4-7
8	Approximately how many patients have you scanned in the last 6 months:	>15	>15	>15	>15	6-10
9	What scans do you typically perform?					
a.	eFAST					✓
b.	Focused cardiac	✓	✓		✓	
c.	IVC volume assessment					
d.	Lung	✓	✓	✓	✓	✓
e.	Procedural	✓	✓	✓	✓	
f.	Other (specify)			REBOA and cannulation procedures		
10	I am confident adjusting the basic knobs such as gain and depth	5	5	4	4	5
11	I am confident at choosing the correct probe for a given patient and purpose	5	5	4	5	5
12	I am confident finding and obtaining vascular access with POCUS	5	4	4	5	3
13	I am confident at performing either a FAST exam and/or an EFAST exam	3	3	2	3	4
14	I am confident that I can diagnose pericardial effusion with POCUS	5	4	3	5	4
15	I am confident that I can diagnose a pneumothorax with POCUS	5	5	4	5	4
16	I am confident that I can diagnose fluid in the peritoneum such as hemoperitoneum or ascites with POCUS	3	4	3	2	4
17	I am confident in my ability to obtain basic cardiac views with POCUS	4	5	3	5	3
18	I am confident in my ability to assess left ventricular function with POCUS	3	5	3	4	3
19	I am confident in my ability to perform a thoracic examination with POCUS	4	4	4	5	4
20	I am confident in my ability to use POCUS for diagnostic neonatal applications	2	2	2	3	1
21	I am confident in my ability to perform neonatal procedures with POCUS	2	2	3	4	1
22	I am confident in my ability to evaluate right ventricle function and volume overload with POCUS	4	4	2	3	3
23	I am confident in my ability to evaluate volume responsiveness with POCUS	4	4	2	2	3
24	I am confident in my ability to diagnose shock with POCUS	4	4	3	4	3
25	I am confident in my ability to acquire images and interpret them with POCUS “putting it all together”	4	4	3	5	4

Years in practice, anesthesiologists (mean, SD): 18 ± 4
