# Supplementary material for: Ascending with ultrasound: telementored eFAST in flight—a feasibility study
Source: Emerg Radiol. 2023 Dec 9;31(1):25–31. doi: 10.1007/s10140-023-02186-x (PMC10830595; doi:10.1007/s10140-023-02186-x)
Supplement: Supplementary file 2 — (PDF 252 kb) [file 10140_2023_2186_MOESM2_ESM.pdf]

Feedback questionnaire, remote expert

Patient no.:
Examiner:
Date: ___/___/___/
Flight altitude:
Weather:

Time, start of exam: ________ Time, end of exam: ________

5	4	3	2	1
Good Diagnostic Quality	Moderate Diagnostic Quality	Fair Diagnostic Quality	Some Grossly Useful Information	No Useful Diagnostic information
e.g., organ parenchymal detail visualized	e.g., can rule out significant hydronephrosis or abdominal fluid	e.g., only gross pathology	e.g. some organ margins visualized	

(*1) Right upper quadrant (Morrison's pouch)* Yes ☐ No ☐

Clear visualization of the junction between the upper pole of the kidney and the liver edge

Rate quality of image obtained:
(Please circle relevant answer)

1 2 3 4 5

(2) Left upper quadrant

Clear visualization of the junction between the upper pole of the kidney and the spleen edge Yes ☐ No ☐

Rate quality of image obtained
(Please circle relevant answer)

1 2 3 4 5

(*3) Pelvis*
a) Clear visualization of the bladder wall and Yes ☐ No ☐

b) Clear visualization of retrovesical or retrouterine space Yes ☐ No ☐

Rate quality of image obtained
(Please circle relevant answer)

1 2 3 4 5

(4) Pericardium
a) Clear visualization of pericardium and myocardium and — Yes ☐ No ☐

b) Clear visualization of myocardium and opposing liver edge — Yes ☐ No ☐

Rate quality of image obtained
(Please circle relevant answer)

1 2 3 4 5

***(**5) Lung ultrasound*
a) Clear visualization of the pleura? — Yes ☐ No ☐

Rate quality of image obtained
(Please circle relevant answer)

1 2 3 4 5

(6) Overall
How confident would you be to confirm the absence of free fluid from the images obtained?
(Please circle relevant answer)

1 2 3 4 5

Were there any technical problems during the scan?
If Yes, please detail in extra comments section.

EXTRA COMMENTS

___

___

___

Feedback questionnaire, examiner

Patient no.:
Examiner:
Date: ___/___/___/
Specialty:
Flight altitude:

(1) How clear and easy to follow did you find the remote expert's instructions?

Very easy Easy Undecided Hard Very Hard

(2) Would you be happy to perform the scan on a real patient if a remote expert was guiding you? (*Please tick your relevant answer*)

Yes	No	*(If no, please give reason for answer in box below)*

(3) Please add any further comments about your experience in the box below (Optional)

